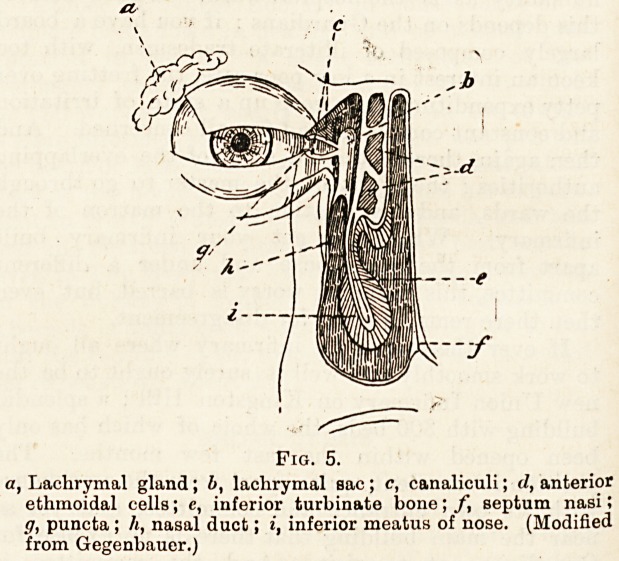# The Hospital. Nursing Section

**Published:** 1903-02-21

**Authors:** 


### The Hospital.

### Nursing Section.

Contributions for this Section of "The Hospital" should be addressed to the Editor, "The Hospital" Nursing Section, 28 & 29 Southampton Street, Strand, London, W.C.

No. 856.—Vol. XXXIII. *SATURDAY, FEBRUARY* 21, 1903.

### Notes on News from the Nursing World.

### THE QUEEN'S NEW BADGE.

One of the most interesting features of the ceremony performed by Queen Alexandra at the Herbert Hospital took place after the opening of the Nurses' Home. It has always been the intention of her Majesty since she became President of the Imperial Military Nursing Service to design the badge for the use of her nurses, and on Monday the gold, silver, and bronze badges presented to the matrons, sisters, and staff nurses were the fulfilment of this intention. There is ultimately to be a special ribbon to accompany the badge, but this is not yet quite ready. Therefore, in order that the distinction should not be incomplete, the Queen, with characteristic thoughtfulness, added in each case a piece of the ribbon which is usually only worn by the ladies of her own household. It is a broad stripe of dark blue, edged on each side with narrow stripes of red, white, and blue, and it will be treasured all the more by the fortunate recipients because of the associations.

### MALE NURSES IN THE ARMY.

We are officially informed that a comprehensive scheme of reorganisation, aiming at a differentiation of the duties of non-commissioned officers and men of the Royal Army Medical Corps, has been under consideration at the War Office. It has been decided to recommend that the non-commissioned officers and men of the corps should be divided into four distinct sections—(1) nursing section, (2) cooking section, (3) clerical section, and (4) general duty section. Special attention has been paid to the training of men for the nursing section, who will in future be employed in nursing duties only. A very complete scheme of nursing instruction is being drawn up, and every effort is being made to develop this section. Her Majesty has signified her wish that the most proficient and best conducted male nurses should be admitted to her nursing service, and a scheme for carrying out this object, including extra remuneration to such selected non-commissioned officers and men, and the granting of a special badge to denote the distinction that has been conferred upon them, is under consideration. To the general duty section will be allotted those duties in hospitals only indirectly connected with the care of the sick. Advancement to a higher grade of orderly and promotion (except to a limited extent in the clerical and cooking sections) can only be obtained through the nursing section. Revised rates of pay are under consideration, with the view of making the nursing section the most important and attractive. It is believed that the employment of specially-selected men in nursing only, with adequate remuneration, will attract to the corps the best class of men, while the admirable courses of instruction, for which officers commanding hospitals and matrons are responsible, should enable men to qualify themselves for posts in civil life on retirement.

### A CERTIFICATE IN CONTEMPLATION.

In addition to the details forwarded to us by the Director-General of the Royal Army Medical Corps, we learn that it is hoped to give the male nurses a certificate after a full course of training and examination. This, taken in conjunction with the other important reforms foreshadowed, should go far towards the attainment of the object which the Queen has in view, namely, that the best of male nurses, no less than the best of female nurses, should be attracted to the service of which she is in a very real sense the head. It has always been an admitted drawback that the orderlies employed in nursing were only allowed to remain in the department for a limited time. In these circumstances they have not had fair play. Now, however, it will be impossible for them to complain that they do not have adequate opportunities of showing their capacity, and, of course, the provision of a proper system of training should make a vast difference to their qualifications for the work. The scheme of instruction and the revised rates of pay promised, will be awaited with much interest.

### THE NURSES' QUARTERS AT THE NEW MILITARY HOSPITAL IN LONDON.

The question of the accommodation for the nursing sisters at the new Military Hospital, opposite Vauxhall Bridge, is still under consideration. When the plans were sanctioned, some two years ago, the sisters' house was designed in accordance with the ideas then in vogue with regard to army nursing; since the inauguration, however, of Queen Alexandra's Imperial Military Nursing Service, those ideas have changed, and a much larger staff than was originally intended will now be drafted to the hospital on the Embankment. Moreover, it is also intended to quarter the reserve staff, to be sent out in cases of emergency or for service in smaller hospitals, in the new building, as well as sick nurses home from foreign stations. The little house, therefore, under the same roof as the quartermaster's residence, with its five bedrooms, two sitting rooms, and kitchen, is totally inadequate to meet the needs of the reorganised nursing service. We understand that as soon as the necessary ground can be secured, a suitable building will be erected.

### FLOWERS FOR SOLDIERS.

The wards of the Herbert Hospital, which at festive seasons are wont to be decorated after

"Tommy's" own heart, and by his own skilful fingers, were on Monday, the occasion of the Royal visit, after strenuous polishing, beautified simply by plants and flowers. This undoubtedly arose from a feeling, prevalent among the sick soldiers, that Queen Alexandra wanted to see just how the wards in which her soldiers were nursed looked every day; and nothing could have been prettier than the bright colours and graceful grouping of the early daffodils, primulas, and daisies, relieved by the broad fan-like palms in huge pots. It is not, perhaps, sufficiently remembered by the public, which is lavish in gifts of flowers to the civil hospitals, that the wards of the great military hospitals need brightening just as much. The soldier who has been on the march, or in the battlefield, feels soothed and cheered in sickness by the sight of the familiar flowers of Old England, and we are sure that gifts of plants and flowers would be gratefully welcomed by the matron of the Herbert Hospital for use in the wards.

### NURSING THE MENTALLY AFFLICTED.

In the interview between our Commissioner and the medical superintendent of Claybury Asylum, Dr. Robert Jones manifests for the movement for improving the position of the asylum nurse, the sympathy which might have been expected from him. The institution of which he is the capable head is one of the most up-to-date in the Kingdom, but he is by no means content with things even as they are at Claybury. He would like to see a nurses' home provided for the nursing staff, and he looks forward to the time when, by rendering the lot of the asylum nurses more attractive and more remunerative, they may be more largely drawn from the ranks of the educated. Meanwhile, in the light of the interview, we are able to make a few practical suggestions for the consideration of the Committee of the London County Council, who are responsible for the government of Claybury Asylum. One is that 6 o'clock, or an hour and a half before breakfast, is an unnecessarily early hour for the nurses to be in the wards in the winter, bearing in mind that they do not leave until 8 in the evening and have no long spell of time off duty. Surely, too, the practice of giving the nurses in their first year only ten days' holiday is a mistake. As Dr. Jones says, it is in the first year that the strain of the work is generally felt most seriously; and therefore the longer the holiday the more likely it is to prove recuperative and beneficial. We observe that the last meal of the day in the asylum is tea at 4.30. Presumably, the members of the staff may buy food when they are out between 8 and 10 in the evening. But suppose they want more tea, is there not a temptation to use a spirit lamp in their rooms? Of course, this is very properly forbidden in the regulations, yet regulations are sometimes disobeyed, and it is quite conceivable that a spirit lamp employed for the purpose of obtaining a cup of tea by a tired nurse late in the evening, might be the cause of a frightful disaster. Dr. Jones has recently effected structural improvements in the buildings with the view of affording additional means of egress in the event of fire. But prevention is better than cure, and one possible cause of fire might perhaps be eliminated if the Committee can see their way to alter the system of rations so as to include a meal of some kind between 4.30 p.m. and 7.30 a.m.

### SHAM CO-OPERATION AT GLASGOW.

In December last, following the annual meeting of the members of the Glasgow and West of Scotland Co-operation of Trained Nurses, we congratulated the two hundred nurses who belong to that organisation on their success in, at any rate, postponing the adoption of a scheme, opposed by the executive, for a new constitution which, if carried, would have the effect of placing them in the position of servants instead of members. We now understand that on Monday next a special general meeting will be held in Glasgow, at which it will again be sought to pass the scheme, and the rules which have since been circulated, make it more clear than before that if the executive have their way, the vital principle of co-operation will be abandoned. In these circumstances we can only strongly advise the members to persist in their attitude of opposition, and we suggest that in view of the fact that the nurses who at the annual meeting voted for delay were subsequently called upon by the executive to apologise, the voting on Monday should be by ballot.

### NURSES AND VOLUNTEER BALLS.

Through the kindness of the Medical Superintendent of the Portsmouth Parish Infirmary, the members of the nursing staff have on several occasions this season enjoyed an evening's dancing. On Friday last twelve of the sisters and probationers received invitations to one of these pleasant balls. These extra privileges have not interfered with the duty, late evening time off being granted and the ordinary breakfast hour being kept. The nurses go in uniform, are properly chaperoned, and they appreciate the courtesy of the President of the Committee of the Sergeants' Mess in extending his hospitality to them.

### A CHANGE OF NAME.

The governors of Northampton General Infirmary have changed the name of that institution to Northampton General Hospital. The principal reason given for the alteration is that in the nursing profession an infirmary is regarded as inferior in status to a hospital. All infirmaries should not be tarred with the same brush, and there are, of course, General and Poor-law infirmaries at which the highest standard of nursing is in force, just as there are hospitals at which a standard below the highest is observed. But the creation of "the qualified nurse" would unquestionably stimulate the mistaken impression that an institution called an infirmary must of necessity be inferior in status to an institution called a hospital.

### GOLD WATCHES FOR SMALL-POX NURSES.

The Romford Guardians have decided to present gold watches to two of the nurses in their service in recognition of the manner in which they attended patients suffering from small-pox. The epidemic started in the workhouse, and the nurses in question were constant and active in ministering to the sufferers from the disease. Obviously it was their duty to be so, but the recognition of special efforts and the exhibition of personal courage in such circumstances is always encouraging.

### KINGSWOOD NURSING HOME.

At a recent meeting of the Warmley Guardians the Kingswood Nursing Home was criticised adversely by a member of the Board. He affirmed that the supposed annual meeting of the Home was held "once in seven or fourteen years, or whenever the authorities thought fit." No nursing organisation which dispenses with annual meetings can expect to retain the confidence of the public; and it appeared on the surface as if Mr. Jefferies had made out an excellent case for opposing a subscription by his Board to the Kingswood Home. Unfortunately for him, however, he was not acquainted with or had overlooked facts. The Kingswood Nursing Association was only started on June 18th last year, and no annual meeting could therefore have been held. As in six months the nurses have attended 188 poor people, and paid 3,326 visits, the actual charge working out at 1d. per visit, it is a matter for regret that such a promising institution should have been disparaged without reason. We hope that the Warmley Guardians will recognise the duty of giving it substantial support.

### ANOTHER OVERWORKED SUPERINTENDENT NURSE.

At a meeting of the Gateshead Board of Guardians, the resignation of the superintendent nurse was announced, and Mrs. Spence Watson, one of the most influential members of the board, said that the action of the superintendent nurse was no doubt due to the large amount of work. She herself, "on visiting the workhouse, had found 63 patients, imbeciles and children, under one nurse." This is a state of things which is not likely to have the effect of inducing nurses to remain in the employ of the Gateshead Guardians, and if, as the chairman affirms, the medical officer has absolute power to increase the nursing staff, the sooner he does so the better.

### THE QUESTION OF TRAVELLING EXPENSES.

The question of travelling expenses has been raised at Exeter. A recently appointed assistant nurse has written to the Guardians, stating that she cannot take up the appointment on account of family troubles. She did not indicate their nature, but as she used blackedged paper they may be presumed. The Guardians were rather annoyed, and have passed a resolution requesting her to return the travelling expenses paid her when she was interviewed. The mover of the resolution suggested that if she did not voluntarily meet their demand, she should be sued as an example to others, and it was also proposed that in future the payment of expenses should be conditional on the position being taken up after appointment.

### A DISTRICT NURSE DISCONTINUED AT LAUNCESTON.

The ancient capital of Cornwall ought to be ashamed of itself. It will not even find the meagre sum of £40 a year for the support of a district nurse. For six years there has been one attached to the Launceston Infirmary and Rowe Dispensary, but in future her services will not be available. It is a matter for regret that when other parts of the Western county have shown their warm appreciation of the value of district nursing, Launceston should be on the side of retrogression.

### THE PROPOSED NORTHAMPTON COUNTY ASSOCIATION.

The Towcester meeting, to which we referred last week, was remarkable, we are informed, for the absence of well-known medical men, the truth being that many of them do not regard the scheme with favour, and are entirely in sympathy with the objections urged by Lady Knightley. We are glad to hear it, for there is all the more reason why the Northamptonshire medical men should do their best, not by mere abstention, but by active effort, to prevent the county from carrying into effect a movement which, on the lines proposed, is quite unworthy of it.

### ABERGELE DISTRICT ASSOCIATION.

In the fifth annual report of the Abergele and Pensarn District Nursing Association, which has just reached us, mention is made of the resignation in October of Nurse Booth, "in consequence of a complete breakdown in health," and of a small presentation to her as "a slight recognition of the thoroughly conscientious and careful way in which she had worked for nearly five years in the town." We are informed that since the report was printed the death of Miss Booth, at the age of 35, has taken place at her home in Worcester. Before her engagement in North Wales she worked in Leeds, and last year she obtained the first prize for the best answer given in The Hospital *Nursing Section* to a question on the nursing of typhoid fever. Her successor at Abergele is doing good work, and we are glad to see that the receipts of the association last year exceeded the expenses by about £17.

### SHORT ITEMS.

General Sir John and Lady French will open a bazaar on April 15th at Deal, in aid of the Victoria Hospital and Private Nursing Institution.—Lord Methuen, in his evidence before the War Commission, referred in terms of approval to the work of the nurses in South Africa.—A performance of "Oberon and Titania" by eighteen English and American children has been given at Rome, at the residence of the American Ambassador, in aid of the Anglo-American Nursing Home.—The late Mr. W. R. Shaw, of Stockport, and formerly of John William Street, Huddersfield, has left a legacy of £1,000 to the Huddersfield Victoria District Nursing Association.—The death at Norwich of Miss Gaze, a veteran nurse of 85, is announced.—On Thursday night last week a representation of "San Toy," a musical play, was kindly provided by Mrs. Carl Meyer and a party of friends for the entertainment of the patients at the National Hospital for the Paralysed and Epileptic, Queen Square, Bloomsbury.—The s.s. *Staffordshire*, which reached Southampton on Monday, had on board Sister A. B. Hill, of the Army Nursing Service Reserve.—Dr. R. A. Young will deliver an address at 10 Orchard Street, W., on Thursday next, at 5.30 p.m., to the members of the Royal British Nurses' Association on "The Heart and its Action in Health and Disease."

### The Nursing Outlook.

" From magnanimity, all fear above;
From nobler recompense, above applause;
Which owes to man's short outlook all its charm."

### INFIRMARY TRIALS.

THE difficulties of infirmary nursing are many and great; the difficulty of securing good nurses for a service that is less attractive than hospital life, and many other well-known trials, have lately been discussed in these pages in connection with the report of the Local Government Board. And the last word has not been said with regard to that report by a long way, and the discussion on it is bound to go on until some wiser means of meeting the lack of suitable nurses is found.

But apart from the securing of good nurses there is the question of retaining good nurses; there is the question of making the atmosphere of the infirmary as fired with nursing enthusiasm and the cause of humanity as is the hospital ward. A good deal of this depends on the Guardians; if you have a board largely composed of illiterate tradesmen, with too keen an interest in a low poor rate, the fretting over petty expenditure may keep up a state of irritation and constant complaint bad for all concerned. And then again, there is the old story of the overlapping authorities; the right of the master to go through the wards, and his relation to the matron of the infirmary. Where you get your infirmary built apart from the workhouse and under a different committee, this form of worry is barred, but even then there remains room for disagreement.

If ever there was an infirmary where all ought to work smoothly and well it surely ought to be the new Union Infirmary on Kingston Hill; a splendid building with 300 beds, the whole of which has only been opened within the last few months. The position is excellent, quite apart from the workhouse with its own entrance and committee, and yet so near the main building that there is no excuse for Guardians not to visit. And the committee is thoroughly interested in its fine building, with its open galleries and phthisis wards, and its *x*-rays room; the chairman, Mr. Andrews, is a constant visitor, and has that kindness of heart and courtesy of manner that make him welcome everywhere, and to rich and poor alike. And for the good of nursing details and little points of domestic economy there is Mrs. Paston Brown on the committee, ever ready to discuss the merits of different flannels, or to consider the number of hours a woman can work, or how many holidays a nurse can have. The matron of the infirmary, Miss Smith, is fully trained, and with experience at Birmingham and Bradford, two of the best schools possible, her one aim and object is to make the new infirmary one of the best training schools for nurses in the kingdom. Dr. Donald, the senior medical officer, aids and abets her in her desire, lectures to the nurses, supplies them with books and diagrams, and presides over their Christmas theatricals. And yet the local papers are full of the difficulties at the infirmary; there have been too frequent resignations amongst the sisters and nurses, and too many of the officials wear a worried look, and there is an obvious sense of strain that makes the work go without the swing and joy that accompanies a satisfactory bit of duty. Now the whole essence of nursing is to secure an atmosphere of rest; it is the work of the medical man or surgeon to institute active remedies where necessary, but the main object of the good nurse is always to secure to her patient that peace and comfort and absence of fret and jar that nature loves, and so to help forward the healing of body and mind. And so the sense of anxiety in hospital work is hurtful to the patient, and no infirmary is at its very best where there is knowledge of difficulties and disagreements to fret the staff. It seems that at Kingston there is a disagreement between the two doctors, and with that disagreement we have nothing to do here whatever in so far as it concerns them, or concerns the Guardians: the origin of it is unknown to us, but there is probably some right on both sides as in most quarrels, and there is probably some very trifling matter at the root of the disagreement which could easily be put right by a few manly words on either side—again, as in most quarrels. But what concerns us here is the weariness of the thought that even when your building and your Guardians and your arrangements are all satisfactory, the slightest thing may arise in a public institution, and disturb the smooth working of the wheels. Because the meetings and the proceedings are public, and the local press takes up the matter, and the nurses talk, and then bicker, and then take sides, and discord replaces harmony. It is very difficult to stop this; it is very difficult indeed, under the circumstances, to retain the spirit of loyalty that ought to run throughout the whole infirmary and prevent any public squabbles. The temptation in the less exciting atmosphere of the infirmary is to decrease discipline. But it is a mistake; every probationer should be taught the etiquette of behaviour towards the whole staff, and the whole staff ought to observe the utmost loyalty in reporting everything concerning the nursing through the matron. On her shoulders rests the burden of the efficient nursing of the institution; she has to select and train the nurses, and her position should be rigorously upheld, and no one should be allowed to question or blame the nurses except herself. Every effort should be made to keep the nurses apart from all disagreements amongst other officials or Guardians; with the politics of the board, as it were, the nurses have no part. This, then, is one of the real trials of infirmary life with which no commission can deal, and the remedy of which lies in the hands of the staff themselves. You cannot keep the reports out of the local papers, and you cannot keep the local papers away from the nurses. But surely a strong effort of loyalty all round would keep the worry and fret away from the women who already have sufficient strain on their lives? Amongst the many chronic and infirm cases that find their way into the infirmary wards kindliness and pleasant manners to ease the long days of pain are just as important as skill in bandaging or a knowledge of therapeutics. No one should be allowed to serve these unlucky remnants of humanity who is not only willing to serve with hand and head, but also willing to serve with the heart. And this applies alike to doctors and nurses.

### Lectures on Ophthalmic Nursing.

By A. S. Cobbledick, M.D., B.S.Lond., Senior Clinical Assistant Royal Eye Hospital, late House-Surgeon and Registrar, Royal Eye Hospital.

### LECTURE IV.—ANATOMY OF THE CONJUNCTIVAL SAC AND EYELIDS—THE LACHRYMAL APPARATUS.

*The Conjunctival Sac* is formed by the reflection of the fine membranous conjunctiva, from the anterior surface of the eye to the upper and lower lids. The shape of the sac is altered by opening and closing the lids. The upper portion of the sac is larger than the lower.

The accompanying diagram, fig. 4, indicates the arrangement of the conjunctiva with reference to the eyeball and

Fig. 4.

*a*, Eyeball; *b*, antrum of Highmore; *c*, optic nerve; *d*, levator palp. supr.; *e*, frontal sinus; *f*, fibrous portion of upper lid; *g*, superior fornix; *h*, tarsal cartilage; *i*, conjunctival sac; *l*, lower lid; *m*, opening of Meibomian gland.

lid, and also the manner in which a slip of tendon of the elevator of upper lid is attached to its upper limit; it thus becomes clear that when the upper lid is raised the relaxed and loose conjunctiva is at once placed on the stretch.

The ocular conjunctiva is quite loosely attached to the sclerotic, but at the cornea it is continued as a fine layer of epithelium, which can only be demonstrated under the microscope. The line of reflection of the conjunctiva from the eye to the inner surface of the lids is called the fornix.

At the margin of the lids the conjunctiva becomes continuous with the skin.

The upper and lower lids are continuous with each other at the inner and outer extremities of the palpebral fissure: these points are called the internal and external canthi.

If the region of the internal canthus is carefully studied, you will observe a small pinkish elevation called the caruncula, on the surface of which can be seen with the aid of a magnifying glass a few fine short hairs; under certain atmospheric conditions, *e.g.* in foggy weather, small particles of foreign bodies are washed on to the caruncle and held there by the hairs.

Immediately to the outside of the caruncula there is a well-marked vertical fold of conjunctiva called the plica semilunaris This fold is of interest, because it is a rudimentary representative of a well-marked structure found in many of the lower animals, viz, the membrana nictitans. The membrana nictitans can be very well demonstrated in a cat, by drawing the hand firmly over the eyes towards the ears.

*The Eyelids.*—These are two movable curtains protective to each eye, and made up of skin fibrous tissue and cartilage.

By personal observation, one can readily see that the upper lid has a wider range of movement than the lower so that when the lids are closed and their edges are in apposition, the cornea is almost entirely covered by the upper lid. If the free margins of the lids are carefully examined, it will be seen that for the most part they are flat, and that from the anterior edge the eyelashes project; along the posterior edge of the lids are the openings—in a single row—of the meibomian glands. Towards the internal canthus the free edge of the lids becomes rounded, and the eyelashes and meibomian glands disappear. At the junction of the flat and rounded portions there is a small raised papilla, at the apex of which is a small perforation called the *punctum lachrymale*; this is the mouth of the canaliculus which gives passage to the tears.

*The Lachrymal Apparatus* consists of:—(1) Lachrymal gland; (2) The puncta; (3) The canaliculi, two in number; (4) The lachrymal sac; (5) The nasal duct.

The lachrymal gland is situated in the lachrymal fossa at the anterior and external portion of the roof of the orbit. A

Fig. 5.

*a*, Lachrymal gland; *b*, lachrymal sac; *c*, canaliculi; *d*, anterior ethmoidal cells; *e*, inferior turbinate bone; *f*, septum nasi; *g*, puncta; *h*, nasal duct; *i*, inferior meatus of nose. (Modified from Gegenbauer.)

portion of the gland projects beyond the orbital margin, and rests upon the eyeball in immediate contact with the conjunctiva, where it is reflected from the lid on to the eyeball.

Passing from the gland to the conjunctival sac, in the region of the upper fornix, are a variable number of very fine ducts, which serve the purpose of carrying the tears, secreted by the gland, to the conjunctival sac; the involuntary blinking of the eyelids causes the tears to wash over the exposed part of the eye and carry any foreign bodies towards the puncta and caruncula.

*The Puncta* are the two small orifices already noticed in speaking of the eyelids; there is one for each lid. It is important to note that the orifices are in contact with the conjuctiva. These are the openings into the

*Canaliculi* or lachrymal canals These are two fine ducts which pass inward to open into the lachrymal sac at about its centre; they are slightly curved, the upper one passing upwards as well as inwards, whilst the lower one has a slight downward curve.

*The Lachrymal Sac* is lodged in the lachrymal groove previously described as lying on the inner wall of the orbit, behind the orbital margin. It forms a *cul de sac* above and communicates below with the nasal duct.

*The Nasal Duct* is that portion of the passage which is contained in the bony nasal cavity; it passes straight downwards and opens into the fore part of the inferior meatus of the nose.

### Nurses' New Quarters at Woolwich: Opening by the Queen.

BY OUR OWN REPRESENTATIVE.

THOUSANDS of people assembled on Monday afternoon outside the Herbert Hospital to see the arrival and departure of the King and Queen; and the entire route, from Well Hall Station to the building itself, was lined with enthusiastic subjects. The winding road was decorated with Venetian masts, flags, and banners, which had a very pretty effect in the bright sunshine, and from the junction of Well Hall Road with Shooters' Hill, up to the gates of the hospital, the masts carried streamers of paper roses and mottoes of welcome. The railway arch just outside the station was hung with red and gold-fringed cloth, and a large banner proclaimed that Woolwich welcomed their Majesties. The Woolwich Borough Council had a grand stand in the station yard, and on alighting from the train a bouquet and an address of welcome, containing an allusion to the memorable visit of Queen Victoria in March 1900, were presented by the Home Secretary (the Right Hon. A. Akers-Douglas) on behalf of the Mayor and Council. Between lines of the 4th Battalion Royal Fusiliers, the Army Service Corps, and the boys of Greenwich Hospital School, all under the command of Colonel J. C. Oughterson, Army Service Corps, the Royal carriage drove, accompanied by a travelling escort of Life Guards, the crowd cheering all the way. The suite in attendance consisted of Captain F. E. G. Ponsonby, the Hon. J. H. Ward, the Hon. Sydney Greville, Lady Lytton and the Hon. Miss Charlotte Knollys. Just outside the hospital were posted detachments of the Royal Horse and Royal Field Artillery. Inside the gates the scene was a bright one. The entrance was draped with flags and carpeted with scarlet cloth, while the covered way leading to the nurses' quarters was hung with red, white and blue. There were flags everywhere: the front of the church was decorated with a trophy of them; they hung from the doors and windows of the married quarters; and enormous standards almost completely covered the front of the corridor connecting the two wings of the hospital.

The band of the Royal Artillery took up its position on the parade-ground, and a guard of honour, formed by the gentlemen cadets of the Royal Military Academy, faced the covered way. The few specially invited guests had seats in the precincts of the church, but as the proceedings were strictly private, the number of these was very small.

About an hour before the arrival of their Majesties a whisper of "Roberts" went round, and the Commander-in-Chief, wearing his cloak, after greeting the chief officers, inspected the guard of honour. A little later, the Adjutant-General (Sir T. Kelly-Kenny), the Quartermaster-General (Sir Mansfield Clarke), the Director-General of the Army Medical Service (Sir William Taylor), Colonel Hutchinson and other officers arrived, with all the members of her Majesty's Nursing Board.

The arrival of the Royal carriage was announced to those within the hospital by the cheering of the crowds, and a Royal salute of 21 guns from the Upper Gun Park; then the King and Queen, having accepted a bouquet and an address from the Borough of Greenwich, in which the hospital is situated, entered the courtyard to the strains of the National Anthem. A third bouquet—of lilies of the valley and Neapolitan violets—was presented, this time by Master Leake, the little son of Colonel Leake, principal medical officer. Their Majesties were received by the Vice-President of the Nursing Board, Countess Roberts, R R.C.; the chairman, the Director-General, A.M.S.; and the members, Sir F. Treves, Bt., K.C.V.O., C.B., Surg.-Gen. A. Keogh, M.D., C.B.; the matron-in-chief, Q.A.I.M.N.S., Miss Sidney Browne, R R.C.; the matrons of civil hospitals, Miss K. H. Monk and Miss M. H. Cave; and the members nominated by her Majesty, the Viscountess Downe and Hon. Sydney Holland; and the secretary, Lt.-Col B. M. Skinner, R.A.M.C.; and by Colonel Leake, P.M.O. for the District; Colonel Whitehead, senior officer; Major Bradell; and Miss Beatrice Jones, the newly appointed matron of the Herbert Hospital.

The Queen wore a black velvet dress trimmed with jet and edged with sable; her cloak and hat were of mauve velvet with silver braid; and the King, who was wearing undress Field Marshal's uniform, appeared extremely cheerful, and quite recovered from his recent indisposition.

First of all, the Queen presented badges as follows:

MATRONS: GOLD BADGES.

The matron-in-chief, Miss Sidney Browne; the principal matron, Miss Becher; the matron of the Herbert Hospital, Miss Beatrice T. Jones.

SISTERS: SILVER BADGES.

Sisters D. V. Briscoe, N. C. Cheetham, S. Lamming, G. E. Larner, L. M. Lyall, G. A. Magill, and W. Potter.

STAFF NURSES: BRONZE BADGES.

Nurses E. M. Bickerdike, A. M. Fitzgerald, S. R. Hughes-Hallett, E. C. Humphreys, E. J. M. Keene, M. Kendall, C. C. R. Moor, M. Pedler, E. M. Pettle, L. A. Rideout, A. A. Wilson, and F. E. C. Watson.

The badges are in the form of a Danish cross enclosed in an oval bearing the words: "Queen Alexandra's Imperial Military Nursing Service." In the centre of the cross is the letter A, and above is the Imperial crown. The reverse of the badge is plain. The ribbon presented by the Queen is that only given by her to the ladies of her own household.

The inspection of the new quarters was then proceeded with, and on arriving on the second floor the uniforms of the various ranks were shown, both the King and Queen being greatly interested.

Crossing the courtyard, their Majesties then went into the wards, the Queen walking with Earl Roberts, and the King with Colonel Whitehead. Lady Roberts followed with Miss Sidney Browne, who was wearing the grey uniform, faced with scarlet, of the Q.A.I.M.N.S., and the order of the Royal Red Cross, the Coronation Medal, the South Africa Victoria and the South Africa King's Medals, the Egyptain Medal, and the Khedival Star.

Two of the surgical wards, into which some medical cases had been carried, were next visited, as well as the operating theatre, which was shown by Sir Frederick Treves. No decorations of any kind were put up in the wards, but some beautiful palms and early spring flowers—daffodils and primulas—were arranged in the centre of each. Very great pleasure was of course given by the Royal visit, especially as some of the soldiers now in hospital had never seen Queen Alexandra before. There were five hundred and fifty-one men in hospital, of whom, however, a good many were convalescent and able to walk about the wards. One hundred and forty-one of the patients had seen service in the South African War.

On leaving the wards the party grouped itself on the parade ground, which was draped with the Royal Standard and the Harp of Ireland, and the King presented medals to a number of orderlies drawn up in line. This over, the band struck up one of Sousa's marches, and their Majesties crossed the yard to the nurses' quarters once more, where tea was

### NURSES' NEW QUARTERS AT WOOLWICH: OPENING BY THE QUEEN—*Continued.*

served to them in the sisters' sitting-room, and to the remainder of the party in the large dining-room. In a few moments the carriages were drawn up, and the Royal visitors drove back to Well Hall Station amid the cheering of the crowds.

The Nurses' New Quarters.

No new building has been erected for the sisters and nurses at the Herbert Hospital, but reappropriation has taken place, what used to be the offices of the secretary-registrar now forming the first floor of the nurses' house. The entrance faces the courtyard, and just inside is on the left a cloak-room, and on the right a small lavatory with hand-basins; the sisters' sitting-room, and a general mess-room and small but cosy writing-room, as well as the kitchens and the servants' hall are on this floor. The first floor is devoted to the matron and sisters, the separate bedrooms of the latter being on either side of a corridor. The staff nurses are on the second floor, with similar rooms, and their own sitting-room. The matron's sitting-room has her own furniture and pictures, but her bedroom contains furniture formerly belonging to Lord Kitchener, and showing signs of honourable service. It is, as might be expected, very plain, and designed rather for use than appearance. The colouring of the corridors and staircases is buff with a dado of chocolate and red, and the bedrooms are mostly papered in canary yellow, while the dining and sitting-rooms are red. A military bias appears in the choice of most of the colouring, which would be improved by the addition of a few good pictures, especially in the sitting-rooms. Preparations have been busily going forward for a month or more, and most zealous help in getting things ready for the opening has been rendered by the sisters. Miss Jones, who arrived at the hospital only some four days before the Royal visit, has had a very busy time, and considering that "men were in every room" on the Thursday previous, it was wonderful to see the transformation effected. Giant palms beautified the entrance, while fresh spring flowers made windows and tables gay and bright for the auspicious occasion. Great assistance in putting the final touches was given by Viscountess Downe and by the matron-in-chief. Flowers were sent by Viscountess Downe, Lady Roberts, Miss Monk, Mrs. Leake, Mrs. Whitehead, and other ladies.

Photographs of the sisters and nurses were taken after Monday's ceremony.

### The Asylum Nurse.

A CHAT WITH THE MEDICAL SUPERINTENDENT OF CLAYBURY: BY OUR COMMISSIONER.

The growing interest of everyone in the care of the mentally afflicted—quickened not a little by the terrible fire at Colney Hatch—and the increasing importance of the elevation of the nursing standard in asylums, suggested a visit to the most modern of the great institutions under the charge of the London County Council. Dr. Robert Jones, the medical superintendent at Claybury, to whom I mentioned the idea, not only raised no objection, but courteously offered to receive me himself, and to afford me any information he could. Many travellers by rail on the Great Eastern railway have seen Claybury Asylum in the distance, owing to its imposing position on one of the Essex hills; but few have any conception of the extent of the institution. Even when the lodge gates on the summit of the hill are reached, there is a drive of about half a mile to the principal entrance, through the most admirably-managed and carefully-kept grounds. It is, in fact, quite impossible to imagine a more suitable situation, or surroundings better calculated to be beneficial to the inmates. As a complete inspection of the vast pile of buildings would mean ten miles of walking under cover, I, of course, only saw a small portion of the interior, yet quite sufficient to enable me to form a fair impression of its general character. In company with the medical superintendent I passed through one of the largest wards containing a number of female patients who were about to have their mid-day meal; the quite ornate chapel, which holds 800 worshippers; the splendid hall in which every week entertainments of various kinds are given to several hundreds of persons; the cheerful work-room, where the women do their sewing; the nurses' commodious sitting-room; the extensive kitchen, in which a staff of some 30 persons, inmates and officers, with a male cook at their head, are employed; and the fine pathological laboratory, under the charge of Dr. Mott and his assistants. This laboratory, Dr. Jones explained, was for the whole of the County of London, and any qualified medical man may work there for three months or more, free of charge, so long as his investigations in the opinion of the director have something to do with the pathology of insanity.

As we completed our little tour, Dr. Jones said that he had read with much pleasure the article in The Hospital nursing section on "The Asylum Nurse," and thought that it would tend to encourage those who were engaged in asylum work.

"You agree, then," I said with the estimate of the special demands made upon the nurses in the way of character and discipline."

"I do entirely," he rejoined, "and I am sure that all efforts which are made with the object of showing the important nature of the services rendered by the asylum nurse, and the need that she should possess the highest possible qualifications, must do good."

The Matron and Staff.

"Before I ask you to give me the benefit of your views in regard to what you consider the most essential qualifications in an asylum nurse, I shall be glad if you will tell me a few particulars about your own nursing staff, and first as to the number."

"Of nurses—all our female attendants are called nurses—we have 150 on day duty and from 20 to 25 on night duty. Of males, who are called attendants, there are about 120. At the head of the nursing staff is a matron who has enjoyed varied and valuable experience. She possesses the certificate of the Medico-Psychological Association, and has been in succession head of the laundry, head of the work-room, head nurse, and assistant matron. She is very tactful and kind to the patients, knows just when to send for the medical officer, sees that the women are properly booted, laced, and have all their clothing tightly fastened and in order. In fact, she acts upon the principle, which in an institution like this must always be recognised, that nothing is unimportant."

The Training.

"Is any distinction made between the nurses?"

"There are two classes, the charge nurses and the ordinary nurses. We have about fifty of the former, who are paid on a higher scale, and who dine at a separate table. Every charge nurse must have the certificate of the Medico-Psychological Association for proficiency in nursing and attending on the mentally afflicted."

"But not the others?"

"Not necessarily, though many have it; and the aim of the Asylum training is to render every nurse fit to be a charge nurse. Although the possession of the certificate is a *sine qua non*, there are nurses who pass the examination of the Association, but who do not become charge nurses because they are not otherwise fitted for the responsibility."

"Do you care to say anything about the training and examination of candidates for the certificate of the Association?"

"Both nurses and attendants must be trained in an institution for the treatment of mental disorders for not less than two years, before they can become candidates for examination. The system of training consists of lectures and demonstrations by the medical staff of the institution; clinical instruction in the wards; exercises under the head and charge nurses in the practice of nursing and attendance; study of the 'Handbook of Nursing,' issued by the Association, and periodical examinations, at least yearly."

### HOURS AND SALARIES.

"What are the hours on duty? and are those of the charge nurses different from those of the others?"

"No, all of them come on duty at 6 A.M., and leave off at 8 P.M."

"When do they breakfast?"

"At half-past seven. Of course I am speaking of Claybury only. There are, I believe, asylums in which the patients do not rise in winter until seven, and in such the day nurses would not be on duty until that hour. All our nurses and attendants have half an hour for breakfast, lunch, and tea, and three-quarters of an hour for dinner."

"As you do not mention supper, I conclude that tea is the last meal. What time is it served?"

"Half past four. As to off duty, the nurses may go out from 8 to 10 P.M. They have a day a week, and the male attendants a day and a half. On the other hand, the attendants have a fortnight's holiday in the year, and the nurses get three weeks, except in the first year, when have only have half the time."

"Is a shortened holiday for the first year in your opinion a wise arrangement?"

"It appears to be considered necessary. But there is no doubt that the first year of nursing here imposes a greater strain than succeeding years; most of the cases of nurses being absent from sickness are those of the non-seasoned, whose environment is new."

"I should like to include something about the rate of pay and any privileges offered."

"The charge nurses and night nurses receive from £25 to £33 per year, and the remainder from £18 to £24. Male attendants of the first class and night attendants receive from £36 to £43, and second-class attendants from £29 to £35. In each case there is an annual addition of £1. All grades join three months' probation, after which time free uniform is given. For nurses it consists of one stuff dress, two print dresses, six collars, six pairs of cuffs, four caps, six aprons, one bonnet, one hood, one shawl, and one belt for keys. These are renewed when necessary, and in most cases annually. Gratuities are given annually to nurses and attendants in the choir."

### THE HOME LIFE.

"What provision is made for the nurses indoors?"

"They have, as you have seen, a very large general sitting-room; and they have also a dining-room of their own. Many have separate bedrooms, but some sleep in dormitories."

"Do you not think that they should all have a separate bedroom?"

"I attach so much importance to the home life of the nurses that I think there ought to be, as at hospitals and many infirmaries, a nurses' home, with separate sitting-room and dining-room for the charge nurses, a library, and, above all, a separate bedroom for each nurse, so that she might always have one place sacred to herself."

### SPECIAL DIFFICULTIES.

"As bearing upon the question of personal qualifications, what are some of the difficulties the nurse has to face here?"

"Some of the patients say the most irritating things, especially at certain periods. All the patients suffering from melancholia are worst in the morning, and if not attended to often try to commit suicide. The neurasthenic patients, on the contrary, are more violent towards the end of the day. It is essential that, however trying they are in these circumstances, the nurse must not have even the least feeling of a desire to retaliate. Of course, a nurse is thrown upon her own resources very much. She is frequently face to face with a patient who is frightfully homicidal; and she has to see to her safety both in regard to herself and others."

"What means are open to her?"

"Not the use of a strait waistcoat. There have never been any strait waistcoats here in the history of the asylum. Sometimes 'seclusion' has to be resorted to. In that event the patient is kept alone in a room bolted from the outside during the day, and when this occurs between 8 A.M. to 6 P.M., it is technically termed "seclusion" and has to be reported to the Lunacy Commissioner. But this is very rarely required. Although we have about 2,400 inmates and some 500 admissions each year, there are never more than six patients in 'seclusion' during the whole year. The greatest of all wants in the nursing of the mentally afflicted is tact, and tact is at no time more indispensable than in inducing the patients to take their food. This and sleep are the essence of the treatment."

### CHRISTIAN WORK.

"The asylum nurse," continued Dr. Jones, "must not think it lowering to do what are called menial duties, though she has to do work which, if it were not Christian work, would be positively repulsive. She must be prepared to perform practically any service. She can exercise the most wholesome influence. For example, if the patients see her sewing, mending, or scrubbing, this encourages them to do the same. It is not easy to exaggerate the mental effect upon the patients of the performance of routine duties, and their conformance to them is the first indication of improvement in their state. The nurse, too, should be a companion as well as a servant. She should sing to the patients, play to them, read to them, and take them out shopping. For these reasons we want educated girls as asylum nurses—girls who can be depended upon for various stimuli to the patients."

### THE HOSPITAL NURSE.

"Have you had any hospital-trained nurses here?"

"Yes; and they have been eminently helpful in our infirmary with acute and bed-ridden cases. They had to be taught their mental work, but I consider that if we could have a combination of the hospital aud the asylum-trained nurse, we should get a perfect system. Unfortunately, the freedom and elasticity to which the hospital nurse is accustomed, in comparison with that of the asylum nurse, makes her feel her duties in an asylum irksome, and she cannot continue. I had a hospital nurse for about five years, but on more than one occasion she had to go away on extended sick leave because she found the work so trying; and at last she gave it up altogether."

### ROOM FOR A BETTER CLASS.

"Do any of your nurses look after male patients?"

"No; I have had no experience of women nursing men, and have not the means of employing them. But I believe that some superintendents are employing hospital nurses with a predilection for mental nursing to nurse acute cases. At Claybury we look chiefly to the improvement of the training of our own nurses, and to attracting a better class of candidates for asylum work. There is plenty of room for a better class, especially in private mental nursing. Several who were trained here are engaged in private work."

"But is not that a loss to you?"

"Undoubtedly. Moreover, it points to a duty on the part of the authorities to render the position of the nurses as attractive and remunerative as possible. In some asylums this has been recognised, and the salaries have been increased, with a bonus after a term of years. You may take it as a last word that I fully admit the importance of making the condition of life for the asylum nurses such that when their experience makes them valuable we can succeed in retaining their services."

### Everybody's Opinion.

### THE COLNEY HATCH FIRE.

"One of the Nurses at Colney Hatch Asylum" writes under date February 13th: Your article of January 31st having only just come under my notice, I feel I cannot let it pass without saying a few words regarding some of the questions that have been put regarding the terrible calamity that has overtaken us here. I am a member of the staff and was present at the fire, and I can speak with truth of what I saw. You ask how it was that 51 of our poor patients were lost and the staff saved; in answer to that I say they faced death fearlessly, led on by the brave heads of this building and all the other officers, who worked till they dropped, those who slept there and lost their all, worked in their night clothes, thinking nothing of their losses or themselves, only their patients, and were at the last dragged out by the firemen. You ask, were the staff assembled and divided into relays under officers, and each charged with a special duty? The only answer to that is, had that been waited for at such a fire the whole of the patients would have been lost instead of 51. We all knew the duty we had to perform, and met on one common ground with our officers to save our patients, fellow creatures; and all, from the head of the building downwards, faced death over and over again, and it is the great sorrow of all here that we could not save them all. Also have you considered the great difficulty we had to face? The chronic cases you speak of were in most cases suicidal and epileptic. The suicidals saw the chance that they had been always watching for, and in spite of all efforts to save them were lost; others flew back for such things as a letter, packet, or orange, and had to be saved twice over. I was amongst those who crawled through the terrible smoke, the doctors and men carrying as many as three patients at a time, one on the back and one under each arm; but we do not wish to speak of these things at all, only I myself say there were many acts of heroism done there that will never be known. As regarding doors, every door that was possible to reach was open, everything that human power could do was done; and there was plenty of water, but fear much was wasted in such a furnace, and, on the other hand, the greater part of our own hose and hydrants were in use, so that the local firemen may have found themselves at a loss. However, they know and we know all the water and firemen in London could not have saved the annexe, but they saved the main building. There is one question I cannot let pass as regards fire practices: they are standing rules here, and always have been more especially at the annexe, where they were always held every fortnight under the supervision of the head doctors, matrons, and other officers, and it was a usual thing to see about once a month, or oftener, the firemen, with their engine, practising in the airing courts, always attended by the medical superintendent and other officers, and sometimes members of the committee. Only a week before the fire I saw the night nurses going to the annexe for their fire practice. That was a beautiful place, with every comfort for the patients and with most stringent rules and every possible precaution against fire. I am not sending these few poor words in any real answer to the questions that were inserted, because for some of them to be answered you must stand as we did in the face of a raging furnace, in half a gale of wind, and have all the difficulties to contend with we did; then, and then only, will the whys and wherefores be truthfully answered and pressed home.

### Appointments.

Aston Union, Birmingham.—Miss Annie Coyne and Miss Elizabeth Mary Woodbridge have been appointed charge nurses. Miss Coyne was trained at the Workhouse Infirmary, Birmingham, where she subsequently became charge nurse, and has been engaged in private nursing. Miss Woodbridge was trained at the Union Workhouse Infirmary, Stapleton, Bristol.

Fountain Fever Hospital, Tooting Grove. — Miss A. S. G. Bryson has been appointed assistant matron. She was trained at Crumpsall Infirmary, Manchester, where she was five years as sister and second assistant matron. She has also been attached to the Co-operative Society for Trained Nurses, Glasgow.

Ilkley Hospital and Convalescent Home.—Miss Jeanie S. Abernethy has been appointed assistant matron. She was trained at the University College Hospital, London, was private nurse for four years at the Leeds Trained Nurses' Institution, and was three years at the Western Fever Hospital, Fulham, S.W.

Jessop Hospital for Women, Sheffield.—Miss E. E. Longworth has been appointed sister. She was trained at the Royal Infirmary, Manchester, and the Royal Infirmary, Bristol, and holds the L O.S. certificate.

Leigh Union Infirmary.—Miss Minnie Hampson has been appointed superintendent nurse. She was trained at the Manchester Workhouse Infirmary.

Paddington Infirmary.—Miss Mary Gertrude Hague has been appointed assistant matron. She was trained at the London Hospital, and has been Queen's nurse at Woolwich, nurse at the South-Western Fever Hospital, London, and staff nurse at the London Hospital.

Queen Alexandra's Imperial Military Nursing Service.—Miss Becher and Miss C. H. Keer have been appointed principal matrons. Miss Becher was trained at the London Hospital, where she was afterwards sister. Miss Keer was trained in the United States, has been a member of the Army Nursing Service since 1887, and nursed in South Africa during the greater part of the war, receiving the decoration of the Royal Red Cross in recognition of her devotion. She has lately been superintending sister at Colchester.

Throat and Ear Hospital, Brighton.—Miss Gladys Geikie Griffith has been appointed staff nurse. She was trained at the Beckenham Hospital, Kent, and has held positions at the City Orthopædic Hospital, Hatton Garden, E.C., and the South Eastern Fever Hospital, London. She has also done private nursing.

Walsall Union Infirmary.—Miss Hannah Davies has been appointed charge nurse. She was trained at Wolverhampton Union Infirmary.

Warrington Union Infirmary.—Miss Mary J. Biggin has been appointed superintendent nurse. She was trained at the Sheffield Union Hospital and was afterwards night superintendent. She has also been charge nurse at the South Eastern Fever Hospital, London.

### Echoes from the Outside World.

### Opening of Parliament by the King.

THE visit of the King and Queen to Woolwich on Monday was followed on Tuesday by the opening of Parliament in person by his Majesty, both events been marked by enthusiastic demonstrations of loyalty on the part of spectators along the respective routes The King, who was accompanied by the Queen, left the quadrangle of Buckingham Palace exactly at half-past one, having been preceded by five other Royal carriages containing members of the Household. The State coach was drawn by eight cream horses, gorgeously caparisoned. In addition to the outriders and the leaders, three attendants walked on each side of the carriage, and the Yeomen of the Guard were in close proximity. The King, who appeared in the best of health, wore a military uniform, and the Queen, seated on his left, was attired in white. They arrived at the House of Lords at two o'clock, five minutes after the Prince of Wales had taken his seat, wearing his robes as a Royal Duke. First in the King's procession were the State functionaries, who made their obeisances to the still vacant Throne, and to the Prince of Wales. Then the King advanced with his Consort, leading her with his right hand, and took his place upon the Throne, the Queen at his side. After the Leader of the House, the Leader of the Opposition, and others had been fetched from the House of Commons, the King read his speech in a loud clear voice, having first donned his cocked hat with plumes. The King's procession returned in the same order as it came; Buckingham Palace was reached again about three o'clock. The principal measures foreshadowed in the King's speech are an Irish Land Bill, an Education Bill for London, and a Bill for regulating the employment of children.

### Memorial to the late Queen of Denmark.

QUEEN ALEXANDRA desires that a memorial should be erected in Copenhagen to the memory of her late mother, Queen Louise. It will be a joint tribute made by King Christian and all the members of the Danish Royal Family, and it will be erected in the rose garden at Bernstorff Castle. The memorial will consist of a portrait of Queen Louise, accompanied by an allegorical figure of Immortality, and it will be fashioned of very beautiful Grecian marble sent for the purpose by the King of the Hellenes. Queen Alexandra has also ordered a casting of Thorvaldsen's celebrated "Christ" in St. Mary's Church, Copenhagen, which is said to be destined for a church in London.

### Mr. Chamberlain in the Dutch Centres.

ON leaving Port Elizabeth the Colonial Secretary visited Graaf Reinet, where he arrived on Friday. The town was decorated, and large crowds welcomed him. In a speech which he delivered in reply to an address, Mr. Chamberlain alluded to the prominent part played in the rebellion by many of the inhabitants of the district; and with regard to the future, he asked why the people of Graaf Reinet should be more disloyal, more anti-British than those of the late Republics of the Transvaal and the Orange River Colony. On Saturday afternoon Mr. Chamberlain reached Middelburg, where a notable speech of welcome by Mr. De Waal, chairmain of the municipal council, and secretary to the South African Bond, was delivered, and an address presented. The Colonial Secretary, in reply, said that he came to Cape Colony as a peacemaker, and he urged that, as the two races had got to live together, they should trust one another. Mr. Chamberlain and his party spent Sunday at Stormberg, passed through Beaufort West and Matjesfontein on Monday, Paarl on Tuesday, and on the evening of that day arrived in Cape Town. At Paarl there was no enthusiasm.

### The Enthronement of the Primate.

THE ceremony of enthroning the Primate took place at Canterbury last week and was of the usual stately and impressive character. An immense congregation assembled in the Cathedral, and the procession in the nave was singularly representative of both Church and State. When Dr. Randall Davidson had complied with the legal formalities, he was placed on his throne by the Archdeacon, and subsequently installed in the chapter-house. At the luncheon in the Cathedral library the Archbishop made a notable speech in reply to the toast of his health, in which he made graceful allusions to his predecessors, and touched on the problems of the Church of to-day. His Grace mentioned that 40 years ago he went, "a shy, awkward little boy, to Harrow, and that among those who from his earliest days showed him the greatest kindness was his venerated friend the Dean, who was seated on his left to-day." With obvious feeling at the close, he asked that he might have the prayers and assistance of all, so that when he was laid on sleep the words might be said that, "He at least tried to serve his generation according to the will of God."

### The President of the United States on Marriage.

PRESIDENT ROOSEVELT has written a letter to Mrs. John Van Vorst, who is part author of a book called "The Woman who Toils." This has been used by the lady as a preface to the publication which appears this week. The President, who has six children of his own, writes very decidedly upon what he calls the crime of "race-suicide." He says: "I do not know whether I most pity or most despise the foolish and selfish man or woman who does not understand that the only things really worth having in life are those, the acquirement of which means cost and effort. If a man or woman, through no fault of his or hers, goes throughout life denied those highest of all joys which spring only from home life, from the having and bringing up of many healthy children, I feel for them a deep and respectful sympathy, the sympathy one extends to a gallant fellow killed at the beginning of a campaign. . . . But the man or woman who deliberately avoids marriage and has a heart so cold as to know no passion, and a brain so shallow and selfish as to dislike having children, is in effect a criminal against the race, and should be the object of contemptuous abhorence by all healthy people."

### The Employment of Barmaids.

THE question of the advisability of abolishing barmaids altogether in the United Kingdom is still exciting attention. It has been proposed that influence should be brought to bear upon licensing magistrates so that they shall take into consideration the possibility of making the granting of licenses conditional upon the gradual discontinuance of the employment of women attendants at the bar where intoxicating liquors are sold, and that five years should be the extent of the notice given. But Lady Frances Balfour pleads that it would be a serious injustice to deprive an enormous number of women of the means of earning a livelihood, many of whom are thoroughly respectable, although she admits that their work is far from being desirable work for women. She asks all who are interested in the question to exercise a little patience before forming an opinion. The Act which has just come into force in England, if discreetly and firmly administered, may, Lady Frances maintains, possibly so far diminish drunkenness that a public-house will be as proper and safe a place for women to serve in as an ordinary shop. Further, she asserts that the proposal that the women should be dismissed gradually is only kind upon the surface. If in London alone 7,832 women were dismissed at one blow, the sympathy of the public might assist them, but if gradually, though the hardship to the individual woman would be as great, the fact would probably pass unnoticed.

### For Reading to the Sick.

"LEAVE ME TO-MORROW."

Sum up at night, what thou hast done by day;
And in the morning, what thou hast to do.
Dress and undress thy soul: mark the decay
And growth of it: if with thy watch that too
Be down, then wind up both, since we shall be
Most surely judged; make thy accounts agree.

In brief, acquit thee bravely; play the man.
Look not on pleasures as they come, but go.
Defer not the least virtue: life's poor span
Make not an ell by trifling in thy woe.
If thou do ill, the joy fades, not the pains:
If well, the pain doth fade, the joy remains.

*George Herbert.*

It is very helpful to make a habit of offering, morning by morning, the troubles of the day just beginning to our dear Lord, accepting His Will in all things, especially in all little personal trials and vexations.

Seek physical calm and quiet. "Wait on the Lord." When you feel utterly cold, chilled to the marrow, lie still at the dear Masters feet; look up, though you cannot see His face; hearken, though no sound meets your ear. That same gracious countenance which beamed on the thirty-five year sufferer at the pool of Bethesda is turned on you, though you have not waited thirty-five years: and the same voice which asked of him, "Wilt thou be made whole?" is asking the like of you. "It is I, be not afraid." "Blessed are they that mourn, for they shall be comforted." Take to yourself the consolation of knowing that you are under God's very special protection; for it is He that thus lays the cross on you. Your sanctification depends on bearing it. Do so, then, in all trustful simplicity and quietude. Try to dwell less upon the actual cross than upon the Hand which lays it on you; seek, ask earnestly, to know what He would say to you by it, what lesson He means you to learn.

It is to be noted how, when Jesus appeared with His Resurrection Body to His disciples, giving them His blessing of peace, He showed them the wounds in His hands and feet, as though to impress on them how it was through suffering that He had entered into His glory and to strengthen them to pass by the like road.—*Sidney Lear.*

Lord, it was well with me in time gone by
That cometh not again,
When I was fresh and cheerful, who but I?
I fresh, I cheerful: worn with pain
Now, out of sight and out of heart;
O Lord, how long?
"I watch thee as thou art,
I will accept thy fainting heart; be strong."

"Lie still," "be strong," to-day; but, Lord, to-morrow,
What of to-morrow, Lord?
Shall there be rest from toil, be truce from sorrow,
Be living green upon the sward
Now but a barren grave to me,
Be joy for sorrow?
Did I not die for thee?
Do not I live for thee? leave Me to-morrow.

*C. Rossetti.*

### Notes and Queries.

**The Editor is always willing to answer in this column, without any fee, all reasonable questions, as soon as possible.**

**But the following rules must be carefully observed:—**

**1. Every communication must be accompanied by the name and address of the writer.**

**2. The question must always bear upon nursing, directly or indirectly.**

**If an answer is required by letter a fee of half-a-crown must be enclosed with the note containing the inquiry, and we cannot undertake to forward letters addressed to correspondents making inquiries. It is therefore requested that our readers will not enclose either a stamp or a stamped envelope.**

*Nurses' Expenses.*

(152) I am a monthly nurse, engaged for the end of March. As I was some distance from the patient, and it was a special case, likely to come off early, I have taken a room close to her house. Can I claim expenses while waiting?—*Prudence.*

Not unless you made a special arrangement with the lady.

*Nurse with Lupus.*

(153) Can you tell me if there is any home where a nurse, suffering from lupus, could stay in return for services whilst having the Light Treatment?—*Nurse.*

We fear not. Perhaps an advertisement might help you.

*Convalescent Home.*

(154) Can you tell me of a home where a poor woman could go for a few weeks, and, if possible, take her little girl, aged nine, with her?—*Nurse Norah.*

Consult List of Convalescent Homes in "Burdett's Hospitals and Charities." Nurses can see it at the office.

*Training at Thirty-eight.*

(155) I should be glad to know where I can get general training. I am just 38?—*L. K.*

You are too old for general training. Why not take maternity training?

*Leaving a Patient.*

(156) I am a maternity nurse, and should like to know what legitimate steps I can take to leave my patient, who is very perverse, before the time of my engagement has expired. I should prefer to act independently of the doctor.

You cannot legitimately break your engagement, and nurses should not act independently of the doctor.

*Lip-reading.*

(157) I should be glad if you would kindly tell me where I can get a book on lip-reading for a friend who became deaf after influenza?—*X.*

Apply to the Director, Training College for Teachers of the Deaf and Dumb, 11 Fitzroy Square, W.

*Pharmacy.*

(158) 1. Will you kindly let me know where in Dublin I could get a certificated training in dispensing? Kindly say if such a certificate would rank with a good one in England, and (2) also what salaries it is possible for lady dispensers to earn?—*Excelsior.*

It is always more satisfactory to write direct to the Secretary the Pharmaceutical Society, 17 Bloomsbury Square, W.C., for information on such matters. 2. From £70 to £100 per annum.

*Date.*

(159) Can *Mrs. H.* be informed when Dr. (Miss) Garrett commenced practice?

Miss Elizabeth Garrett—now Mrs. Garrett Anderson—took the degree of M.D. at Paris, in 1866.

**Standard Nursing Manuals.**

"The Nursing Profession: How and Where to Train." 2s. net; post free 2s. 4d.
"A Handbook for Nurses." (Illustrated). 5s.
"Nursing: Its Theory and Practice." (New Edition.) 3s. 6d.
"Nursing in Diseases of Throat, Nose, and Ear." 2s. 6d.
"Surgical Ward Work and Nursing." (Revised Edition.) 3s. 6d. net; post free, 3s. 10d.
"Art of Massage." (Second Edition.) 6s.
"Elementary Physiology for Nurses." 2s.
"Elementary Anatomy and Surgery for Nurses." 2s. 6d.
"Practical Handbook of Midwifery." 6s.
"Mental Nursing." 1s.
"Art of Feeding the Invalid." 1s. 6d.

All these are published by the Scientific Press, Ltd., and may be obtained through any bookseller or direct from the publisher, 28 and 29 Southampton Street, London, W.C.

## Figures and Tables

**Fig. 4. f1:**
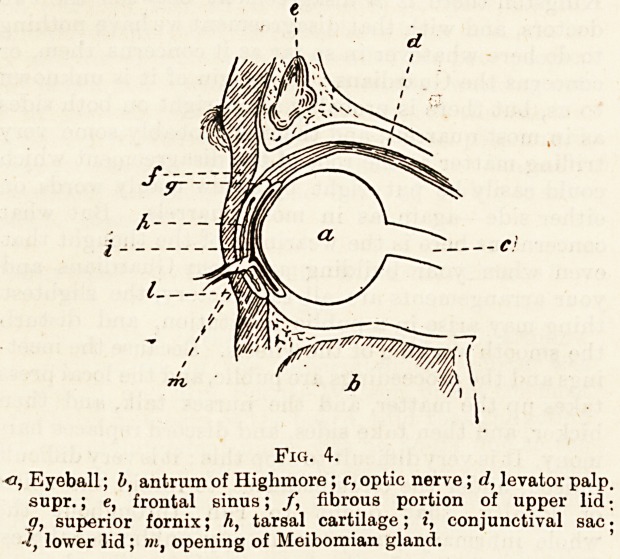


**Fig. 5. f2:**